# Modulation of histone tail electrostatic potentials in nucleosome core particles by acetylation and PARylation

**DOI:** 10.1073/pnas.2511507122

**Published:** 2025-07-21

**Authors:** Nicolas Bolik-Coulon, Philip Rößler, Michael L. Nosella, Tae Hun Kim, Lewis E. Kay

**Affiliations:** ^a^Department of Molecular Genetics, University of Toronto, Toronto M5S 1A8, ON, Canada; ^b^Department of Chemistry, University of Toronto, Toronto M5S 3H6, ON, Canada; ^c^Department of Biochemistry, University of Toronto, Toronto M5S 1A8, ON, Canada; ^d^Program in Molecular Medicine, Hospital for Sick Children Research Institute, Toronto M5G 0A4, ON, Canada

**Keywords:** NMR, post-translational modifications, solvent paramagnetic relaxation enhancement, histone tail–DNA interactions

## Abstract

Cellular DNA is wrapped about an octamer composed of four histone proteins forming the fundamental unit of chromatin structure, the nucleosome core particle (NCP). The intrinsically disordered tails of the histones serve as scaffolds for binding an array of proteins that regulate the fidelity of the genome and gene expression. A variety of posttranslational modifications (PTMs) on the tails have been characterized, including some that alter their overall charge; however, per-residue changes in tail electrostatic potentials for different PTMs have not been reported. Here, using a solution NMR approach in which enhancements of transverse relaxation rates of tail amide and methyl group protons are quantified through the addition of paramagnetic cosolutes, we examine how acetylation and PARylation modulate histone tail electrostatic potentials. Notably, even though both PTMs decrease the net positive charge carried by each tail, their electrostatic potentials either increase or decrease in a tail-specific manner relative to an unmodified NCP. A simple model of tail–DNA interactions is presented to explain these results.

Electrostatics play critical roles in many biochemical processes by modulating the kinetics and thermodynamics of biomolecular interactions, regulating the propensity for phase separation, influencing molecular structure and stability, and impacting catalysis (1, 2). One example is provided by the nucleosome core particle (NCP), consisting of ~147 bp of DNA wrapped around a histone octamer formed by two of each histone: H2A, H2B, H3, and H4 (Fig. 1*A*) (3). All four histones possess a structured domain, which collectively interact to form the core of the NCP, and an N-terminal disordered tail (H2A additionally contains a C-terminal unstructured tail, H2A-C). The stability of the complex relies on electrostatic interactions between the negatively charged DNA (~−300*e*, where *e* is the elementary charge) and the positively charged histone octamer (~+144*e*), with the tails containing disproportionate numbers of lysine and arginine residues (4). In a recent study, we showed that the per-residue near-surface electrostatic potentials (*ϕ_ENS_*) of the NCP tails are negative, despite the abundance of positively charged amino acids in the tails, demonstrating the strong influence of the DNA on tail electrostatics (5). Here, we explore how tail electrostatics and interactions with nucleosomal DNA are modulated by posttranslational modifications (PTMs) that are expected to disrupt charge-based interactions. One such modification, impacting all four histones, is acetylation of the ε-amino group of lysine sidechains, which neutralizes their positive charge (Fig. 1*B*). This epigenetic mark regulates binding of effectors to chromatin (4), modulates chromatin phase separation propensity (2), and controls transcription and the DNA damage response (6). A second PTM, poly-ADP-ribosylation (PARylation), involves the addition of long, possibly branched, chains of ADP-ribose onto various amino acid sidechains, with each ADP-ribose subunit containing two negatively charged phosphate groups (Fig. 1*C*). Previous NMR studies by our group demonstrated that PARylation of *Drosophila melanogaster* NCPs in vitro occurred exclusively at Ser 10 and Ser 28 of H3 and that roughly 70 ADP-ribose subunits were linked onto each H3 molecule, corresponding to ~140 subunits and an additional charge of −280*e* per NCP (7). This PTM also plays a role in the DNA damage response by serving as a recruitment signal for the DNA repair machinery (8). Both marks increase nucleosome sliding (9), nucleosomal DNA ligation rates and susceptibility to nucleases in vitro (7, 10), and enhance the dynamics of the modified histone tail (7, 10).

**Fig. 1.**
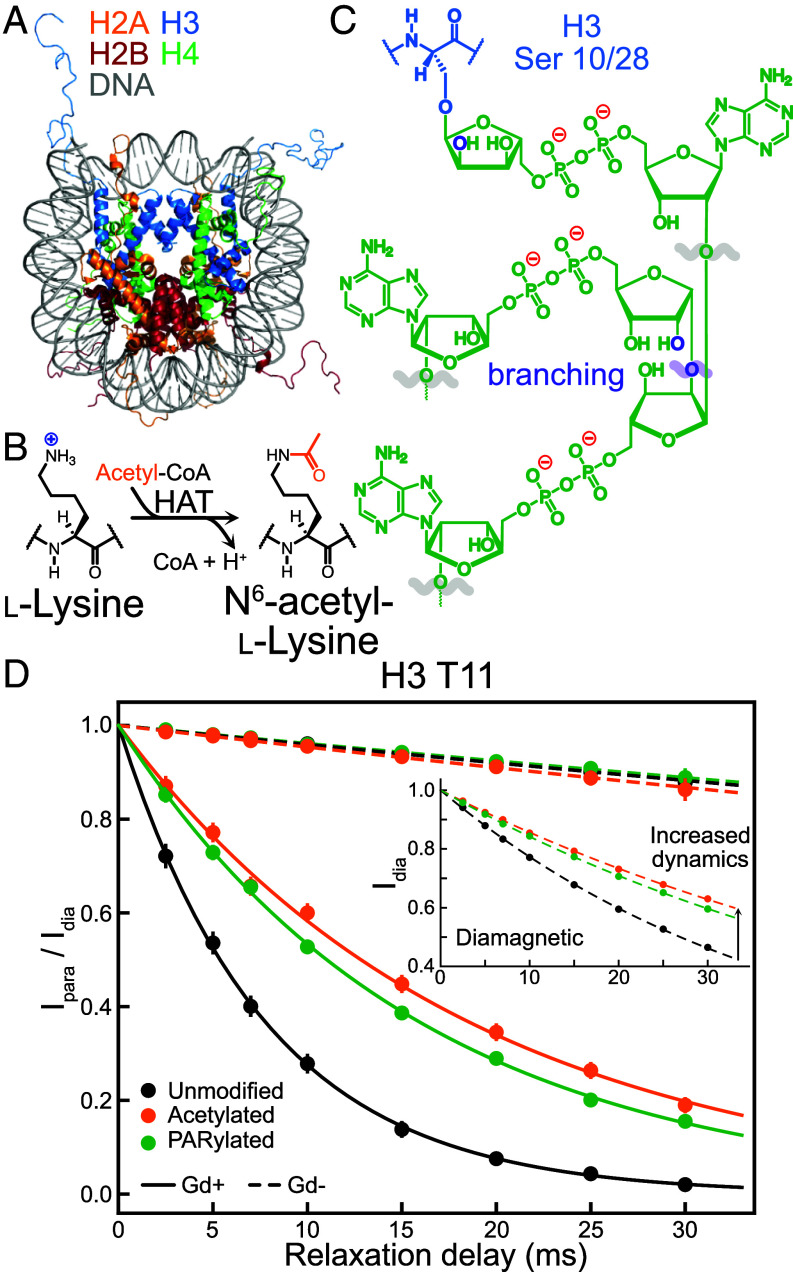
Measurement of sPREs in NCPs with PTMs. (*A*) Structure of an NCP (PDB ID: 1KX5) (11) with histones color coded as indicated on the figure. (*B*) Lysine residues on histone tails can receive an acetyl group (orange) from acetyl-CoA in a reaction catalyzed by a histone acetyltransferase, thereby neutralizing the side-chain charge. (*C*) Schematic of a PAR chain attached to either Ser 10 or Ser 28 of H3. The polymer can be elongated in a linear (gray) or branched (purple) fashion, with branching site or potential branching site oxygens colored in purple. (*D*) Representative I_para_/I_dia_ profiles, that report only on the sPRE contribution to the decay of magnetization, for the amide proton of H3 T11 of the unmodified, H3-acetylated, and H3-PARylated NCP. Although different concentrations of Gd+ and Gd- cosolutes were used to record the experimental data, in this case, 0.3 mM and 6 mM for Gd+ and Gd-, respectively, I_para_/I_dia_ ratios have been normalized to cosolute concentrations of 1 mM so that the effects of the PTMs can be more easily visualized. Inset shows decay profiles for the amide proton of H3 T11 in the absence of cosolute.

The importance of both acetylation and PARylation to chromatin structure, function, and fidelity, and the role of charge in both cases, underscore the need to quantify how tail *ϕ_ENS_* values and tail structural dynamics are affected by these PTMs. Since both PTMs lead to an overall increase in the net negative charge of the tails, either indirectly through elimination of positive charge on lysines or directly through addition of negatively charged ADP-ribose units, it might be expected that the electrostatic potentials of the affected tails would become more negative relative to unmodified NCPs. Here, using a recently developed NMR method (12), we measured *ϕ_ENS_* values of either acetylated or PARylated histone tails of NCPs demonstrating that, in contrast to expectations, the changes in potential can be either positive or negative, depending on the strength of the nucleosome DNA–histone tail contacts in the absence of the PTM.

## Results and Discussion

### PTMs Affect Histone Tail *ϕ_ENS_* Values.

Per-residue electrostatic potentials of tail residues in NCPs prepared with *D. melanogaster* histones and Widom 601 DNA (7, 10) were obtained by recording the decay of transverse magnetization from both tail backbone amide and sidechain methyl protons in the absence of and upon addition of gadolinium complexes that are similar in structure but have different net charges of either +1*e* (Gd+) or -1*e* (Gd−) (13). Fig. 1*D* shows representative intensity decay profiles of the amide proton signal of T11 of histone H3 (H3 T11) that report on the solvent paramagnetic relaxation enhancement (sPRE) contribution from the addition of Gd+ or Gd- cosolutes, for unmodified (black), acetylated (orange), or PARylated (green) H3 tails of NCPs. The obtained rates can be used to calculate site-specific surface electrostatic potentials (12) as these relate to the distribution of the charged cosolutes around the residue of interest (14). In the case of H3 T11, the sPRE obtained from Gd+ decreases upon acetylation and PARylation of H3, relative to the unmodified NCP (Fig. 1*D*), consistent with a less negative *ϕ_ENS_* value at this site upon addition of either of the PTMs. The inset to Fig. 1*D* shows decay profiles measured without cosolute. Notably, in this case, both PTMs on H3 result in decreased H3 T11 decay rates relative to the unmodified NCP that is a signature of increased amplitude ps-ns timescale H3 tail dynamics and consistent with ^15^N spin relaxation experiments showing that both acetylation and PARylation of a given histone tail increase its motion (7, 10).

**Fig. 2.**
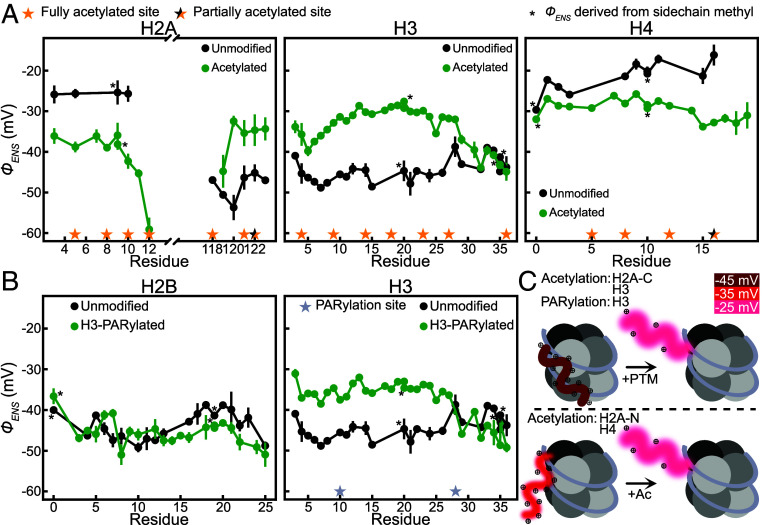
PTMs can have large effects on tail electrostatic potentials. (*A*) Comparison of per-residue *ϕ_ENS_* values for unmodified (Reprinted with permission from ref. 5. Copyright (2025) American Chemical Society) (black) and acetylated (green) tail residues of H2A (*Left*), H3 (*Middle*), or H4 (*Right*). Tail potentials for the histones indicated at the top of each panel are reported; these were also the histones that were acetylated. Sites of full or partial acetylation are indicated by yellow or black and yellow stars, respectively (10). Potentials derived from sidechain methyls are indicated by a * symbol next to the data point. Points are connected with solid lines for visual clarity. (*B*) Effects of H3 PARylation on *ϕ_ENS_* values of H2B and H3. The sites of PARylation on H3 are indicated by the gray stars. (*C*) Schematic rationalizing the effects of PTMs on tail potentials and structural dynamics. Increased tail blurriness indicates larger amplitude dynamics, + symbols highlight lysine or arginine sites, the former of which become neutral upon acetylation, and tail potentials are color coded as indicated. The two scenarios considered can explain the changes in potentials for H2A-C and H3 (*Top*) or for H2A-N and H4 (*Bottom*) upon the addition of PTMs.

Fig. 2*A* shows profiles of *ϕ_ENS_ vs.* residue for NCPs with acetylation of one of histones H2A, H3, or H4 for tail residues of the modified histone (green). The extent of modification at each lysine, established via NMR (10), is indicated by the star symbols along the x-axis. H2B was not considered here as the acetylation was incomplete for most of the lysine sites such that multiple weak peaks were observed for many residues, complicating the analysis (10). Shown also are the profiles derived for tails from unmodified NCPs (black). As discussed previously (5), the unmodified tails can be grouped into two categories: Those with *ϕ_ENS_* values between −40 mV to −50 mV (H2A-C, H2B, H3) and those with potentials on the order of −20 mV to −30 mV (H2A-N, H4). Notably, tails with large, negative *ϕ_ENS_* show reduced potentials (i.e., less negative) when acetylated (notwithstanding H2B for which potentials were not measured for the acetylated tail), with the opposite effect occurring for tails starting from smaller potentials in the unmodified particles. Fig. 2*B* highlights the significant changes in H3 tail potentials arising from PARylation, leading to less negative *ϕ_ENS_* values, as observed for acetylation. In contrast, H3-PARylation results in almost no change of *ϕ_ENS_* values for residues from the H2B tail. In a previous study we showed that H3-PARylation significantly enhances the amplitude of H3 tail dynamics, with little effect on the tails from other histones (7). This lack of change is consistent with the correspondingly small differences in per-residue *ϕ_ENS_* values observed for H2B (rmsd of 4 mV between PARylated and unmodified H3). Taking the 4 mV deviation for H2B tail residues as an upper bound estimate of the experimental error, it is clear that the larger |*Δϕ_ENS_*| values measured for positions on the other tails (10 to 15 mV) reflect bona fide PTM-induced changes.

### A Model for Histone Tail Structural Dynamics.

Fig. 2*C* shows a model rationalizing the observed changes in tail *ϕ_ENS_* values from the NCP modifications. Two scenarios are illustrated. In the first case, the tail of interest interacts strongly with nucleosomal DNA in the absence of a PTM, resulting in large, negative *ϕ_ENS_* values (top; dark red tail). Acetylation or PARylation of the tail would be expected to increase its negative potential in the absence of any structural change. Indeed, Poisson–Boltzmann calculations of isolated tails show that acetylation of those lysine residues observed to be modified experimentally changes tail *ϕ_ENS_* values by ~−20 mV (*SI Appendix*) (15). However, counteracting this effect is the decrease in interactions between tail and DNA that leads to a repositioning of the tail ensemble further from the nucleosomal DNA, increasing per-residue potentials (i.e. *ϕ_ENS_* becomes less negative; top, light red). The latter effect is dominant for acetylation of H2A-C or acetylation/PARylation of H3, since *ϕ_ENS_* values become less negative upon the addition of the PTMs (Fig. *2* A and *B*). In the second scenario, the position of the tail is further from the DNA in the unmodified NCP so that the starting *ϕ_ENS_* is less negative. Concomitant with the removal of positive charge from lysines due to acetylation is the movement of the tail away from the DNA (medium red to light red, bottom). The net effect, as observed for the H2A-N and H4 tails (Fig. 2*A*), is more negative potentials (i.e., removal of charge from lysines is the dominant effect in this case). The resulting *ϕ_ENS_* values are similar to those for the modified H2A-C and H3 tails. In all cases, the amplitude of the dynamics of the tail harboring the PTM increases relative to an unmodified NCP (7, 10), consistent with a tail ensemble that is positioned further from the core upon modification. Such movement may help facilitate the binding of effectors to tail regions that are now more available for interactions. Notably, both the positive *Δϕ_ENS_* values for H3 and the absence of significant changes to *ϕ_ENS_* values for H2B upon H3-PARylation are consistent with rigid PAR-chains that extend away from the tails (16), making little contact with them.

Our results highlight how PTMs can modulate both tail surface electrostatic potentials and tail structural dynamics, providing a mechanism by which charge can be used to regulate the plethora of biochemical interactions that are necessary for proper chromatin function.

## Materials and Methods

The 601 Widom DNA sequence, the histones derived from *D. melanogaster* with or without PTMs (acetylation or PARylation), and the resulting NCP samples were produced as described previously (7, 10). sPRE rates were measured at 37 °C using published pulse schemes (13) and are reported in (15). Further details are provided in extended methods in *SI Appendix*.

## Supplementary Material

Appendix 01 (PDF)

## Data Availability

Numerical data have been deposited in Zenodo (10.5281/zenodo.15643879) (15).
