# Supplementary material for: Modulation of histone tail electrostatic potentials in nucleosome core particles by acetylation and PARylation
Source: Proc Natl Acad Sci U S A. 2025 Jul 21;122(30):e2511507122. doi: 10.1073/pnas.2511507122 (PMC12318224; doi:10.1073/pnas.2511507122)
Supplement: Supplementary file 1 — Appendix 01 (PDF) [file pnas.2511507122.sapp.pdf]

Supporting Information for:

Modulation of Histone Tail Electrostatic Potentials in Nucleosome Core Particles by Acetylation and PARylation

Extended Methods

All NCP samples were produced as described in detail previously.[1,2] NMR samples comprised 20 mM sodium phosphate, 5% D_2O, pH 6.0 at typical NCP concentrations between ~60 μM – 90 μM. An ionic strength (IS) of 120 mM equivalent NaCl is calculated based on the concentration of buffer used, although it is likely slightly higher (effective IS ~ 130 mM NaCl) due to the salt that accompanies the core particle in solution.[3] sPRE rates were measured at 37°C on a 18.8 T Bruker Avance III-HD (acetylated H4) or a 23.5 T Bruker Avance NEO spectrometer (all other samples), both equipped with *x,y,z* gradient TCI cryoprobes. In all PTM-based NCP samples, only one histone was isotopically labeled as $^{2}H,^{15}N,^{12}C$ with specific labeling of $^{13}C^{1}H_3$ ILV methyl groups, while other histones were NMR-silent ($^{2}H,^{14}N,^{12}C$).[1,2] Gadolinium complexes,[4] carrying either an overall +1*e* (Gd-DOTAM-BA, concentrations of 0.1, 0.2 and 0.3 mM; Macrocyclics, Inc.) or -1*e* (Gd-DOTA, concentrations of 2, 4 and 6 mM; Macrocyclics, Inc) charge were used to obtain sPRE rates.

As discussed in detail previously, ϕ_{ENS} values are calculated from the relation:[5]

$$\phi_{ENS} = \frac{-k_B T}{(q_i - q_j)e} \ln\left(\frac{\Gamma_{2,i}}{\Gamma_{2,j}}\right)$$

where q_i = 1, q_j = -1, k_B is Boltzmann's constant, T is the absolute temperature, and $\Gamma_{2,i}$ and $\Gamma_{2,j}$ are the sPRE values in the presence of the positive or negative cosolute, respectively. sPRE values are calculated as the difference in ^{1}H transverse relaxation rates, R_2, in the presence and absence of cosolute (*i.e.*, $\Gamma_{2,k} = R_2(q_k) - R_2(\text{no cosolute})$), and normalized for the differences in concentration between positive and negative cosolutes. The ϕ_{ENS} values reported in Figure 2 were calculated as averages of the potentials obtained from pairs of (Gd+,Gd-) concentrations: (0.1,2)mM, (0.2,4)mM, and (0.3,6)mM. The reported errors are the maximum of {the standard deviation of the 3 potentials, errors in each potential based on uncertainties in measured relaxation rates}. In Figure 2 potentials based on measurements of methyl proton relaxation rates (given as averages over values for both isopropyl methyls of Val and Leu) are indicated with *. Gd concentrations were quantified from calibration curves relating measured water longitudinal relaxation rates to cosolute concentration as described previously.[4]

To evaluate how acetylation affects per-residue near surface electrostatic potentials of isolated tails we have performed computations using the APBS program on tails without and with acetylation.[6,7] The structures of the isolated tails were built in PyMOL,[8] and included five extra glycine residues added to their C-termini (or to the N-terminus of the C-terminal H2A tail, H2A-C) to remove the effect of the terminal carboxyl group (or amino group for H2A-C), with acetyl groups added to the N^{ζ} position and force fields modified to include the acetyl moiety. The desired Poisson-Boltzmann (PB) potentials, ϕ_{PB}, were calculated from

$$\phi_{PB} = -\frac{k_B T}{2e} \ln\left(\sum_{i=1}^{N} \rho_i r_i^{-6} \exp\left(-\frac{e\phi_i}{k_B T}\right) \Big/ \sum_{i=1}^{N} \rho_i r_i^{-6} \exp\left(\frac{e\phi_i}{k_B T}\right)\right)$$

where, e is the elementary charge, ϕ_i is the potential calculated from APBS at grid point i, r_i is the distance between grid point i and the ^{1}H nucleus in question and ρ_i is set to 1 or 0 depending on whether the grid point is accessible (1) to the cosolute or not (0); further details are provided

elsewhere.[4] As a cross validation for calculated ϕ_{ENS} values we have also replaced acetylated lysine residues with glutamine and repeated the electrostatic calculations; very similar values were obtained for the two types of modifications.

Parameters for acetyl-lysine are not included in the APBS package, but are available at http://pc164.materials.uoi.gr/dpapageo/amberparams.php, and have been deposited in a Zenodo file (10.5281/zenodo.15643879) for convenience. Incorporation of these parameters requires modification of multiple scripts (pdb2pqr/aa.py python script, pdb2pqr/dat/AA.xml and pdb2pqr/AMBER.DAT files) that are also included in the Zenodo file. Included also are all measured proton R_2 values, sPRE rates (for each cosolute concentration), and ϕ_{ENS} values for both modified and unmodified NCPs.

References

1. Nosella, M. L. *et al.* Poly(ADP-ribosyl)ation enhances nucleosome dynamics and organizes DNA damage repair components within biomolecular condensates. *Molecular Cell* **84**, 429-446.e17 (2024).
2. Kim, T. H. *et al.* Correlating histone acetylation with nucleosome core particle dynamics and function. *Proceedings of the National Academy of Sciences* **120**, e2301063120 (2023).
3. Bolik-Coulon, N., Rößler, P. & Kay, L. E. NMR-Based Measurements of Site-Specific Electrostatic Potentials of Histone Tails in Nucleosome Core Particles. *J. Am. Chem. Soc.* (2025) doi:10.1021/jacs.5c01567.
4. Yu, B., Bolik-Coulon, N., Rangadurai, A. K., Kay, L. E. & Iwahara, J. Gadolinium-Based NMR Spin Relaxation Measurements of Near-Surface Electrostatic Potentials of Biomolecules. *J. Am. Chem. Soc.* **146**, 20788–20801 (2024).
5. Yu, B., Pletka, C. C., Pettitt, B. M. & Iwahara, J. De novo determination of near-surface electrostatic potentials by NMR. *Proceedings of the National Academy of Sciences* **118**, e2104020118 (2021).
6. Baker, N. A., Sept, D., Joseph, S., Holst, M. J. & McCammon, J. A. Electrostatics of nanosystems: Application to microtubules and the ribosome. *Proceedings of the National Academy of Sciences* **98**, 10037–10041 (2001).
7. Jurrus, E. *et al.* Improvements to the APBS biomolecular solvation software suite. *Protein Science* **27**, 112–128 (2018).

8. Schrödinger, LLC. The PyMOL Molecular Graphics System, Version 1.8. (2015).
